# Regional Anaesthesia Techniques in Liver and Kidney Transplantation: A Narrative Review in Donors and Recipients

**DOI:** 10.4274/TJAR.2026.262509

**Published:** 2026-08-28

**Authors:** Alessandro De Cassai, Burhan Dost, Serkan Tulgar, Bahadır Çiftçi, Sandeep Diwan, Yavuz Gürkan

**Affiliations:** 1 University of Padua, Department of Medicine (DIMED), Padua, Italy; 2 Institute of Anaesthesia and Intensive Care, University Hospital of Padua, Padua, Italy; 3 Ondokuz Mayıs University Faculty of Medicine, Department of Anaesthesiology and Reanimation, Samsun, Türkiye; 4 Bahçeşehir University Faculty of Medicine, Göztepe Medicalpark Hospital, Department of Anaesthesiology and Reanimation, İstanbul, Türkiye; 5 İstanbul Medipol University Faculty of Medicine, Department of Anatomy, İstanbul, Türkiye; 6 İstanbul Medipol University, Mega Hospital, Department of Anaesthesiology and Reanimation, İstanbul, Türkiye; 7 Sancheti Hospital, Clinic of Anaesthesiology, Maharashtra, India; 8 Koç University Faculty of Medicine, Department of Anaesthesiology and Reanimation, İstanbul, Türkiye

**Keywords:** Fascial plane blocks, kidney transplantation, liver transplantation, pain management, regional anaesthesia

## Abstract

Advances in surgical techniques and perioperative care have driven increasing interest in regional anaesthesia as part of multimodal analgesia strategies. In liver and kidney transplantation, however, the adoption of regional techniques remains limited due to concerns regarding altered coagulation, cardiovascular instability, and graft perfusion. This narrative review aims to synthesize current evidence on the feasibility, safety, and clinical utility of neuraxial and fascial plane blocks in both transplant recipients and living donors. A narrative review of the literature was conducted, focusing on regional anaesthesia techniques used in transplantation. The review integrates anatomical considerations, sources of postoperative pain, patient-specific comorbidities, and clinical evidence related to neuraxial anaesthesia and ultrasound-guided fascial plane blocks, including erector spinae plane block, quadratus lumborum block, transversus abdominis plane block, modified thoracoabdominal nerve block, external oblique intercostal block, and emerging techniques. Neuraxial anaesthesia can provide effective analgesia in carefully selected transplant patients, particularly in living donors, but its use is constrained by dynamic coagulation abnormalities and hemodynamic instability, especially in recipients. Fascial plane blocks offer a favorable risk-benefit profile, providing effective somatic and, in some cases, visceral analgesia while preserving hemodynamic stability and minimizing bleeding risk. Evidence supports their opioid-sparing effects and feasibility across a range of transplant procedures. Regional anaesthesia represents a valuable component of multimodal analgesia in liver and kidney transplantation when tailored to surgical incision patterns, sources of pain, and patient-specific physiological considerations. Fascial plane blocks, in particular, appear well suited to transplant populations and may facilitate enhanced recovery while maintaining graft-protective priorities.

**Main Points**

• Regional anaesthesia techniques, particularly fascial plane blocks, provide effective opioid-sparing analgesia for patients undergoing liver or kidney transplantation.

• The use of regional techniques can facilitate early extubation and support enhanced recovery after surgery protocols in the transplant population.

• Clinicians should carefully consider the timing of block performance and the patient’s coagulation status, especially in the context of liver transplantation and potential graft dysfunction.

• Standardization of regional anaesthesia protocols for transplant surgery is still needed, requiring further large-scale prospective clinical trials.

## Introduction

Advances in surgical practice have shifted operative techniques from open surgery toward laparoscopic and robotic approaches and have prompted parallel evolution in anaesthetic and analgesic strategies.^1^ Traditional neuraxial techniques are increasingly being shifted toward, or replaced by, ultrasound-guided fascial plane blocks that provide procedure-specific, mechanism-based analgesia.^2^ Improved understanding of pain pathways and surgical anatomy has facilitated the development of regional anaesthesia techniques targeted at the source of postoperative pain, supporting enhanced recovery and opioid-sparing perioperative care.^3^

Research continues to clarify the selection of appropriate regional techniques for specific surgical procedures, and experience with techniques such as erector spinae plane block (ESPB), rectus sheath block, and ilioinguinal block has contributed to a surgery-specific approach to regional analgesia.^4^

Despite their growing use, regional anaesthesia techniques remain underutilized and insufficiently described in transplant recipients.^5^ This population is characterized by critical illness, complex physiology, and heightened concern for graft perfusion and survival. Consequently, anaesthetic management often prioritizes hemodynamic stability and organ protection, potentially limiting the adoption of regional techniques.

This narrative review aims to synthesize current evidence on regional anaesthesia techniques in patients undergoing liver and kidney transplantation, including both living donors and transplant recipients.

### Search Strategy

As this manuscript was designed as a narrative review rather than a systematic review, a formal protocol-driven study selection process was not undertaken. We chose this approach because, in contrast to a systematic review, which uses a predefined protocol and an exhaustive study-selection framework to answer a specific question, this narrative review aims to provide a clinically oriented synthesis of current evidence.Nevertheless, to enhance transparency, we consider it important to report the literature search strategy we employed. The authors performed a structured literature search in PubMed/MEDLINE, Scopus, EMBASE, and Web of Science using combinations of free-text terms, keywords, and relevant MeSH terms related to regional anaesthesia and transplantation. Search terms included “regional anaesthesia,” “neuraxial anaesthesia,” “epidural,” “spinal anaesthesia,” “fascial plane block,” “ESPB,” “quadratus lumborum block (QLB),” “transversus abdominis plane block (TAPB),” “modified thoracoabdominal nerve block through the perichondrial approach (M-TAPA),” “external oblique intercostal block,” “liver transplantation,” “kidney transplantation,” “living donor hepatectomy,” and “donor nephrectomy.” We aimed to include articles published in any language addressing kidney or liver transplantation in adult and pediatric populations that evaluated the efficacy of any regional anaesthesia technique compared with another regional anaesthesia technique or with standard care. Given the narrative nature of this review, no restrictions were applied to study design: randomized controlled trials, prospective and retrospective cohort studies, case series, and case reports were considered eligible. In addition, all studies potentially relevant to the topic and identified through the bibliographic search were reviewed. Publications were included according to their relevance to the topic, with emphasis on studies addressing anatomical rationale, feasibility, safety, analgesic outcomes, and perioperative recovery in transplant donors and recipients. Reference lists of selected studies were also reviewed for additional relevant articles. The literature search was last updated on 21 January 2026.

Unless otherwise explicitly stated, references to “transplantation” refer to both donors and recipients, while “transplant recipients” refers to recipients of liver and kidney transplants.

As this manuscript is a review of existing literature and does not involve original research on human subjects or animals, institutional ethics committee approval was not required.

### Potential Comorbidities of Interest and How They may Affect Regional Anaesthesia

### • Impaired coagulation and hemorrhage risk

### End-stage renal disease (ESRD)

These patients may exhibit qualitative platelet dysfunction. Uremic patients commonly develop a bleeding diathesis that is primarily related to abnormalities of primary hemostasis, particularly defective platelet function and impaired platelet-vessel wall interactions.^6^ The dysfunction is due to increased intracellular cyclic adenosine monophosphate levels, reduced thromboxane A_2_ production, increased synthesis of prostacyclin, and abnormalities in von Willebrand factor.^7^

Moreover, many patients continue to receive heparin or exhibit residual anticoagulant effects despite guidelines recommending against its use in patients with impaired coagulation.^8^

### End-stage liver disease (ESLD)

Patients with ESLD have historically been considered to be at high risk of hemorrhagic complications; however, it is now well recognized that they are also prone to thrombotic events, reflecting the complex and rebalanced nature of hemostasis in this population.^9^ Typical laboratory abnormalities include thrombocytopenia, prolongation of prothrombin time and activated partial thromboplastin time, and reduced plasma fibrinogen levels.

Thrombocytopenia is caused by hypersplenism secondary to portal hypertension, bone marrow suppression caused by toxic exposures, reduced thrombopoietin production, and immune-mediated platelet destruction.^10, 11^

Alterations in platelet function have also been described in ESLD, but the available evidence remains conflicting, with studies reporting severe platelet dysfunction, paradoxical platelet hyperreactivity, or mixed phenotypes, leaving the true clinical impact of qualitative platelet abnormalities incompletely understood.^12, 13^

Additional caution is warranted in patients with portal hypertension, as engorgement of epidural and paravertebral venous plexuses may increase the risk of bleeding complications during neuraxial techniques.^14^

### • Increased risk of local anaesthetic systemic toxicity (LAST)

### ESRD

Patients with ESRD are at an increased risk of LAST due to altered protein binding, metabolic derangements, and impaired drug handling. Uremia and chronic metabolic acidosis reduce the binding of local anaesthetics to plasma proteins, increasing the free, pharmacologically active fraction in the circulation.^15^ In addition, reduced renal clearance promotes the accumulation of active metabolites of certain local anaesthetics, particularly lidocaine and prilocaine.^16^ Electrolyte abnormalities, especially hyperkalemia and hypocalcemia, and uremic cardiomyopathy lower the threshold for arrhythmias and cardiovascular instability, thereby increasing the likelihood that toxic plasma concentrations will result in severe neurologic and cardiac manifestations.^17^

### ESLD

Patients with ESLD are at increased risk of LAST primarily because of impaired hepatic metabolism and reduced plasma protein binding. Loss of functional hepatocyte mass and decreased hepatic blood flow lead to prolonged elimination half-life and higher systemic drug exposure. Hypoalbuminemia and reduced α1-acid glycoprotein levels further increase the unbound, active fraction of local anaesthetics in plasma.^18^ Finally, cirrhotic cardiomyopathy, hyponatremia (and other electrolyte imbalances), and autonomic dysfunction predispose these patients to exaggerated myocardial depression and malignant arrhythmias once toxic concentrations are reached, increasing the severity of both neurologic and cardiovascular manifestations of LAST.^19^

### • Cardiovascular impairment

### ESRD

Cardiovascular disease is highly prevalent and includes uremic cardiomyopathy,^20^ left ventricular hypertrophy, ischemic heart disease, and heart failure, all of which increase the likelihood of severe hypotension after sympathetic blockade from spinal or epidural anaesthesia. Autonomic neuropathy, particularly in diabetic patients,^21, 22^ can further blunt compensatory hemodynamic responses. Electrolyte abnormalities, especially hyperkalemia, metabolic acidosis, and volume overload, increase the risk of arrhythmias and alter physiologic responses to local anaesthetics.^23^

### ESLD

Cirrhotic cardiomyopathy is characterized, in patients with cirrhosis, by reduced cardiac contractile responsiveness to physiological and pharmacological stress, diastolic dysfunction, and electrophysiological abnormalities, despite the absence of overt structural heart disease.^24^ It is caused by chronic systemic vasodilation, altered β-adrenergic signaling, and increased circulating cardiodepressant substances, all of which impair myocardial contractility and stress adaptation.^25^ All these alterations render these patients particularly susceptible to profound, poorly tolerated hypotension following spinal or epidural sympathectomy.

### Surgical Incisions

Surgical incision is a major determinant of postoperative pain severity and analgesic requirements in solid organ transplantation. In both liver and kidney transplantation, the choice of surgical incision is driven by the need to achieve adequate operative exposure and vascular control, while simultaneously influencing the extent of abdominal wall disruption, neural injury, and postoperative recovery.

In adult liver transplantation, wide upper abdominal incisions are most commonly employed. The classic Mercedes-Benz incision consists of bilateral subcostal incisions joined by a short vertical midline extension below the xiphoid process. This approach provides excellent exposure of the suprahepatic vena cava and hepatic hilum, but is associated with extensive muscle division and bilateral intercostal nerve involvement.^26^ A related alternative is the Chevron (rooftop) incision, which comprises bilateral subcostal incisions without a vertical midline component. Although slightly less disruptive to midline structures, the Chevron incision still involves broad dissection of the upper abdominal wall.^27^

In selected cases, particularly in living-donor liver transplantation or limited recipient procedures, a J-shaped subcostal incision, formed by a unilateral subcostal incision with a short cranial midline extension, may be used to provide adequate surgical exposure while limiting bilateral abdominal wall trauma.^28^ Less commonly, unilateral subcostal (Kocher-type) incisions may be employed for restricted re-exploration or specific donor procedures, resulting in reduced surgical trauma but more limited exposure.^29^

In addition, several modified incisions have been described for specific clinical scenarios. Inverted T (cruciate) incisions, combining bilateral subcostal incisions with a longer midline extension, are occasionally used in multivisceral transplantation or complex reoperative settings.^29^ Hockey-stick or reverse L-shaped incisions, which extend a unilateral subcostal incision inferolaterally, are frequently reported in living-donor hepatectomy to balance exposure with abdominal wall preservation.^29^ Combined upper and lower abdominal approaches, such as a Mercedes incision with an additional Gibson extension, may be required in simultaneous liver-kidney transplantation.

**Figure 1:**
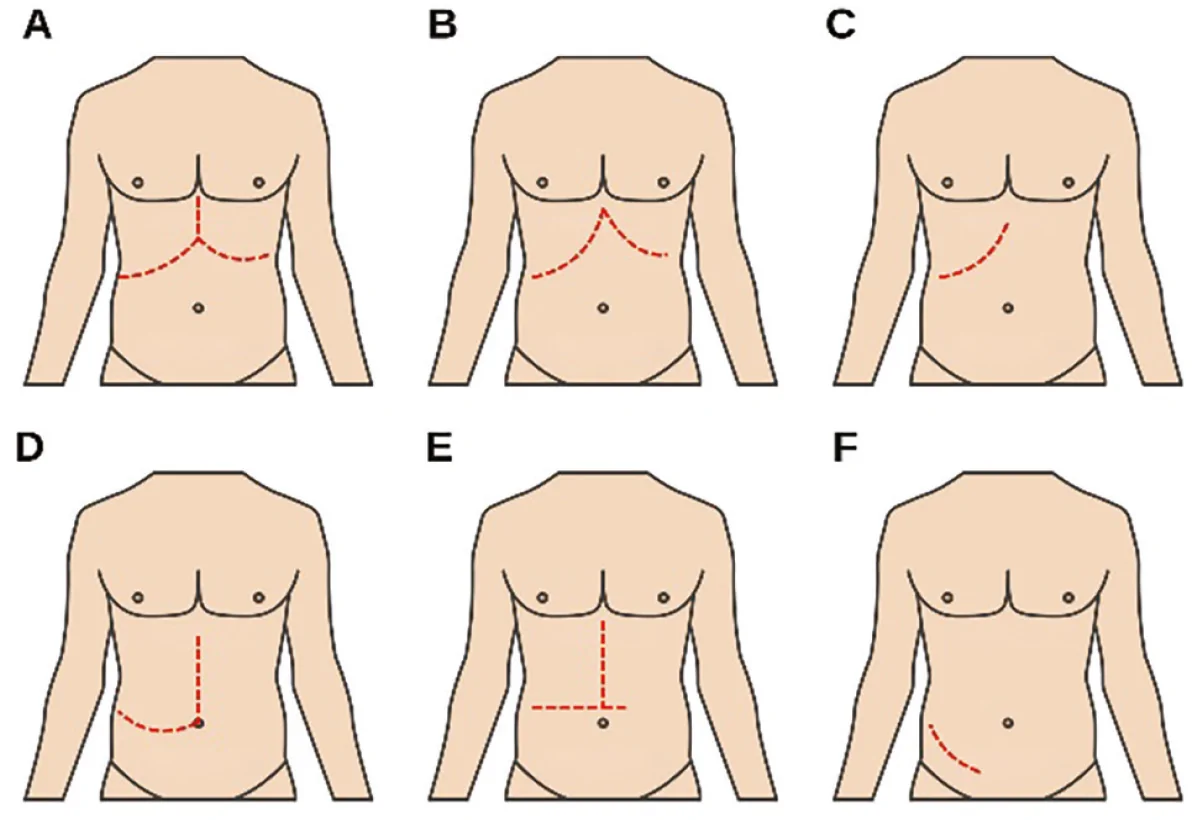
Surgical incision types used in adult liver and kidney transplantation. (A) Mercedes-Benz incision, consisting of bilateral subcostal incisions joined by a short vertical midline extension below the xiphoid process. (B) Chevron (rooftop) incision, composed of bilateral subcostal incisions without a vertical midline component. (C) Kocher incision, a unilateral subcostal incision providing limited upper abdominal exposure, used in selected liver procedures. (D) Makuuchi incision (J-shaped or reverse L configuration), combining a unilateral subcostal incision with a vertical midline extension, frequently used in living-donor hepatectomy and selected liver transplantation cases. (E) Inverted T (cruciate) incision, consisting of a long midline incision with bilateral subcostal extensions, typically reserved for complex, preoperative, or multivisceral transplantation. (F) Gibson incision, an oblique lower abdominal extraperitoneal incision commonly used for kidney transplantation.

In contrast, kidney transplantation is typically performed through a lower abdominal extraperitoneal approach. The Gibson incision, an oblique incision made parallel to the inguinal ligament in the iliac fossa, is the standard approach. This incision allows direct access to the iliac vessels and bladder while avoiding entry into the peritoneal cavity. Variants, such as the Rutherford-Morrison incision, extend the Gibson approach cranially to improve exposure in obese patients or in cases of complex vascular anatomy.^30^ Although smaller than liver transplant incisions, these lower abdominal approaches traverse multiple muscle layers and are closely related to the ilioinguinal, iliohypogastric, and genitofemoral nerves. Figure 1 shows the types of incisions used in transplantation surgery.

### Source of Pain in Kidney and/or Liver Transplantation

In both transplant recipients and living donors, postoperative pain after liver or kidney transplantation has both somatic and visceral origins. The somatic component is generally easier to anticipate, as it arises directly from the surgical incision, drain sites, and trocar or camera port entry points.^31^ These nociceptive inputs are transmitted primarily by the thoracoabdominal, subcostal, iliohypogastric, and ilioinguinal nerves through their anterior and lateral cutaneous branches, depending on the location and extent of abdominal wall trauma. However, abdominal wall innervation is not strictly segmental.^32^ Owing to frequent interconnections and overlap between adjacent nerves and fascial planes, targeting individual nerves in isolation is often insufficient. Instead, fascial plane blocks, which allow the spread of local anaesthetic across multiple neural pathways, are particularly well-suited to interrupting the somatic component of pain in transplant surgery. In addition to incisional nociception, postoperative discomfort in transplant surgery may arise from muscle retraction, sustained tension, and altered abdominal wall mechanics, which may not be perceived as sharp pain but as tightness, pressure, or discomfort. From an anatomical perspective, the ilioinguinal and iliohypogastric nerves, which contribute to the motor innervation of the lower abdominal wall musculature, may play a role in this component of postoperative distress.

In liver transplantation, visceral pain is mediated primarily through the celiac and hepatic plexuses.^33^ The principal sources of visceral nociception include incision or stretching of the hepatic capsule, trauma to surrounding perihepatic tissues, diaphragmatic irritation, and manual manipulation of the gallbladder, biliary structures, and hepatic hilum. Irritation of the diaphragmatic peritoneum may result in referred shoulder pain transmitted via afferent fibers of the phrenic nerve.^34^ Although the anatomical sources of visceral nociception are similar, the relative contribution of these mechanisms differs between living donors and transplant recipients. In living liver donors, partial hepatectomy involves direct incision of the hepatic parenchyma and the Glisson capsule, whereas the remaining innervated liver tissue remains with the patient, resulting in a more pronounced, localized visceral pain component.^35^ In contrast, the transplanted liver in recipients is denervated, thereby limiting direct capsular and parenchymal nociceptive input; visceral discomfort in this context is more commonly related to ischemic and inflammatory stimuli, including ischemia-reperfusion injury, and is often perceived as a diffuse, systemic sensation of pain and fatigue rather than as focal visceral pain.^35^

Visceral innervation of the kidney is primarily mediated through the renal plexus, with afferent fibers originating from the T10-L1 spinal segments and traveling alongside the sympathetic nerves.^36^ Compared with that of the liver, renal visceral innervation is anatomically less complex and more segmentally organized, resulting in a relatively more predictable pattern of visceral pain. In living kidney donors, the entire organ is removed together with its fibrous capsule; therefore, renal capsular nociceptive input is effectively eliminated, and postoperative visceral discomfort primarily originates from manipulation of surrounding retroperitoneal structures rather than from the kidney itself.^37^ In kidney transplant recipients, the transplanted kidney itself is not a direct source of visceral pain, as the organ is denervated and its capsule does not share afferent connections with the recipient. Instead, visceral discomfort instead arises from surgical manipulation of recipient tissues, including the iliac vessels, ureterovesical anastomosis, bladder wall, and surrounding pelvic and retroperitoneal structures. From a visceral perspective, kidney transplantation in both living donors and recipients is generally associated with a less pronounced visceral pain component than nephron-sparing procedures such as partial nephrectomy, in which the kidney and its capsule remain in situ and continue to generate capsular and parenchymal nociceptive input.

Although the visceral component may play a relatively minor role in liver transplant recipients, kidney transplant recipients, and living donors compared with other abdominal or renal procedures, it remains clinically relevant and cannot be disregarded. In addition to surgical tissue injury, pain in this population is further influenced by non-surgical sources, including arterial cannulation, urinary and nasogastric catheters, central venous access, and other invasive monitoring devices, all of which contribute to the overall pain burden.

### Neuraxial Blocks

Neuraxial anaesthesia is not the gold standard for kidney or liver transplantation. Nevertheless, an increasing body of evidence supports its feasibility and safety in carefully selected patients, especially in living-donor kidney and liver transplantation settings.^38^ This evolution reflects a shift from a historical contraindication toward selective use in specific patients.^38^

Several potential benefits of neuraxial anaesthesia have been proposed for patients undergoing kidney and liver transplantation. Although the available literature—particularly large randomized controlled trials—remains limited, recent reviews suggest that these theoretical advantages may be maximized when coagulation status and hemodynamic parameters are carefully optimized.^39^ In this regard, thromboelastography (TEG) has increasingly been adopted as a point-of-care modality for real-time assessment of coagulation during transplantation^40^ and may facilitate individualized hemostatic management. However, to date, no specific evidence or clinical guidelines support using TEG to guide placement or removal of neuraxial catheters in transplant recipients.^41^

### Living kidney donor

In living-donor nephrectomy, neuraxial anaesthesia, in the context of multimodal analgesic techniques within enhanced recovery pathways, may reduce postoperative opioid use and improve recovery; moreover, epidural analgesia has been shown to provide effective pain control, early ambulation, and increased patient satisfaction.^42, 43^ Moreover, in a retrospective cohort of patients neuraxial anaesthesia has been also associated with a significantly lower incidence of delayed graft function in recipients, suggesting a potential protective effect on early graft performance (Table 1).^44^

### Kidney recipient

In carefully selected patients with acceptable coagulation, combined spinal-epidural or spinal anaesthesia has been associated with stable hemodynamics and effective analgesia in renal transplant populations.^38, 45^ However, cohort data indicate that a subset of patients may still require conversion to general anaesthesia.^45^These effects may translate into clinically relevant improvements, as observed in other surgical settings, including better respiratory function, earlier mobilization, and enhanced recovery.^46, 47, 48, 49, 50, 51^ Although direct evidence in kidney transplantation remains limited, these benefits may support the rationale for considering neuraxial anaesthesia as a component of perioperative management in selected kidney transplant recipients, particularly those at increased risk of postoperative pulmonary, cardiovascular, or opioid-related complications (Table 1).

### Living liver donor

Patients undergoing living liver donation may experience a high degree of postoperative pain, which has been reported to be greater than that observed in patients undergoing comparable hepatic resections for malignant disease.^52^ The coagulation status following donor hepatectomy is complex and characterized by a paradoxical profile: conventional coagulation tests often indicate hypocoagulability, whereas TEG may demonstrate a sustained hypercoagulable state persisting up to postoperative day 10.^53^ This imbalance—driven by reduced anticoagulant activity alongside preserved or increased procoagulant factors—predisposes donors to thrombotic complications such as portal vein thrombosis and deep vein thrombosis, despite elevated international normalized ratio values.^53^ Consequently, TEG may better inform decisions than conventional tests do regarding the initiation and duration of postoperative thromboprophylaxis in these patients.^41^

Epidural analgesia has been shown to provide superior,^54^ or at least equivalent^55^ postoperative pain control and improved pulmonary function in living liver donors compared with intravenous patient-controlled analgesia, without a significant increase in complications in retrospective cohort studies (Table 2).

### Liver Recipient

Liver transplant recipients frequently benefit from TEG assessment, as these patients exhibit complex and dynamic coagulopathies that are not adequately captured by standard laboratory tests.^56^ However, TEG has good specificity for hypocoagulability—patients identified as hypocoagulable are at increased risk of bleeding—but limited sensitivity; a normal TEG does not reliably exclude the risk of bleeding. These risks may be further exacerbated in patients with portal hypertension, who can bleed despite apparently normal coagulation parameters due to the presence of extensive collateral circulation and engorged epidural veins.

However, some retrospective series, encompassing hundreds of patients at centers offering epidural analgesia during liver transplantation, suggest that neuraxial techniques may be administered in carefully selected individuals, although the benefit in terms of pain score reduction in these patients remains uncertain (Table 2).^57^

### Fascial Plane Blocks

Fascial plane blocks may offer a more favorable risk-benefit profile anaesthesiathan neuraxial anaesthesia for kidney and liver transplantation. In fact, fascial plane blocks are performed in anatomically superficial planes, distant from the neuraxis, where bleeding is more likely to be clinically apparent and amenable to compression or conservative management.^58, 59, 60^

Moreover, fascial plane blocks largely avoid the sympathectomy, which may adversely affect perfusion of newly transplanted organs, especially during the vulnerable reperfusion phase.^61^

### Liver transplantation: donor and recipient

ESPB is a paraspinal fascial plane block. Its proposed mechanism involves cranio-caudal spread of local anaesthetic to the paravertebral and epidural spaces, resulting in both visceral and somatic analgesia.^62, 63^ ESPB can be performed at any vertebral level; however, when used for liver transplantation, it is most commonly administered between T7 and T9.^31, 39, 64^

Clinical studies in living liver donors and recipients have shown that ESPB reduces postoperative pain scores and opioid consumption compared with standard analgesic regimens.^65, 66^ Continuous ESPB has demonstrated postoperative opioid requirements comparable to those of intrathecal morphine, while providing lower pain scores at 48-72 hours and a reduced incidence of opioid-related adverse effects, such as nausea, vomiting, and pruritus.^67^ In pediatric liver transplantation, continuous ESPB has been associated with lower opioid consumption and earlier return of bowel function.^68, 69^ To date, no ESPB-related complications have been reported in liver transplantation, and complications related to ESPB overall remain exceedingly rare.^70^ QLB is performed between the psoas major and the quadratus lumborum muscles.^64^ Following injection, the local anaesthetic spreads anteriorly and cranially through the thoracolumbar fascia to reach the paravertebral region, providing somatic and visceral analgesia across the T7-L1 dermatomes.^31, 71^

A randomized controlled trial on laparoscopic hepatectomy suggests that QLB may provide effective postoperative analgesia than opioid-based analgesia alone.^72^ In a retrospective cohort of transplant recipients, QLB has also been linked to shorter extubation times, reduced pain scores, and lower intraoperative blood transfusion requirements.^73^ Although no complications have been reported in liver transplant patients, caution is advised because the block is deep, particularly in patients with coagulation abnormalities. In addition, the anatomical proximity to the kidney and liver poses a theoretical risk of organ injury, and motor block may occur secondary to paravertebral spread.^74^

TAPB is performed by injecting local anaesthetic into the fascial plane between the transversus abdominis and internal oblique muscles.^64, 75^ TAPB provides somatic analgesia only, covering the T6-T10 dermatomes, without visceral analgesic effects.^31^ Multiple approaches have been described, resulting in variable spread. In liver transplantation, the subcostal approach may be preferable to the lateral approach because it reportedly provides better coverage of the upper abdominal incision.^31^

Both single-shot and continuous catheter-based techniques have been described in this population^78^anda meta-analysis of three studies demonstrated that TAPB provides effective postoperative analgesia.^76, 77^ To date, no TAPB-related complications have been reported in liver transplant patients.

The M-TAPA is performed at the costal margin of the 9^th^ or 10^th ^intercostal space.^79^ This technique blocks both the anterior and lateral branches of the thoracoabdominal nerves (with more intense blockade of the anterior branches), providing dermatomal coverage from T5 to T12.^31^ This distribution is consistent with the typical surgical approaches used in liver transplantation, including upper midline and lateral abdominal incisions.^31^

In living liver donors, M-TAPA has been associated with reduced opioid consumption, lower pain scores, a decreased need for rescue analgesia, and fewer opioid-related side effects compared with control groups.^80^ Case series of liver transplant recipients have also demonstrated effective analgesia with this technique.^81^ A notable advantage of M-TAPA over ESPB and QLB is that it can be performed in the supine position, eliminating the need for patient repositioning.^31, 80, 81^

External oblique intercostal plane block (EOIB) is performed by injecting local anaesthetic into the plane between the external oblique muscle and the ribs at the 6^th^-9^th^ costal levels.^82^ EOIB targets both the lateral (predominantly) and the medial branches of the thoracoabdominal nerves, providing sensory coverage from T6 to T11.^83^ This block is particularly useful for providing analgesia for lateral abdominal incisions commonly used during liver transplantation.^84^ Depending on the incision pattern, EOIB may be used as a standalone technique or combined with other blocks, such as M-TAPA or TAPB.

Comparative studies have shown that EOIB provides analgesia comparable to that of subcostal TAPB, with similarly low opioid requirements and pain scores.^85^ Case reports and small series support its efficacy in both donors and recipients, including when continuous catheter techniques are used.^86, 87, 88^ Combined approaches, such as EOIB with rectointercostal block, have also been reported to provide effective postoperative analgesia in living donor surgery.^89^ No EOIB-related complications have been reported to date, although caution is warranted regarding the potential risk of pneumothorax due to anatomical proximity.

Although emerging evidence, as discussed above, suggests potential benefits for these patients, at present no major guidelines formally recommend the routine use of fascial plane blocks for patients undergoing liver transplantation, primarily because of limited studies and heterogeneity of results (Table 2).

### Kidney transplantation: donor and recipient

Effective postoperative in kidney transplantation requires coverage of the T10-L1 dermatomes (Table 3).^31, 90^

In renal transplantation, TAPB is most often used as part of a combined anterior abdominal wall strategy, rather than as a standalone technique, due to its limited midline coverage^31, 90^ and its combined use with recuts sheath block have been proposed by some authors. Clinical a randomized study demonstrates that this combination provides analgesia comparable to epidural analgesia, with similar quality-of-recovery scores and a lower incidence of side effects^91^ while other studies show that it can be effectively incorporated into multimodal analgesic regimens for renal transplant recipients.^92^ No TAPB-related complications have been reported following renal transplantation.

Anterior QLB provides combined somatic and visceral analgesia via paravertebral spread of local anaesthetic, with dermatomal coverage typically extending from T7 to L1, making it well suited to renal transplantation.^31, 71, 90^

Multiple clinical studies have demonstrated that anterior QLB significantly reduces postoperative pain scores and opioid consumption compared with either local infiltration alone or systemic analgesia alone among renal transplant recipients and donors.^93, 94^ Continuous catheter techniques have also been successfully employed, particularly in pediatric recipients, resulting in sustained opioid-sparing effects.^95^ QLB has additionally been incorporated into enhanced recovery after surgery pathways for donor nephrectomy, and novel formulations such as liposomal bupivacaine have been reported in selected pediatric cases.^96, 97^

Among fascial plane techniques, anterior QLB may provide superior analgesic efficacy compared with ESPB in renal transplantation, particularly with respect to opioid reduction.^98^

No complications have been reported following QLB in renal transplant patients. Nevertheless, as a deep block, QLB warrants caution in patients with coagulopathy.^90^

ESPB is a paraspinal fascial plane block that can be performed at the T8-T10 levels for renal surgery.^90^

Clinical studies indicate that ESPB reduces postoperative opioid consumption following renal transplantation compared with standard analgesic regimens.^99^ Case series have also reported effective analgesia in living-donor nephrectomy.^100^ However, comparative data suggest that anterior QLB may offer superior analgesic efficacy and greater opioid-sparing effects than ESPB.^99^ No ESPB-related complications have been reported following renal transplantation.

The quadro-iliac plane block (QIPB) is a novel fascial plane technique performed at the level at which the quadratus lumborum muscle attaches to the iliac crest. Local anaesthetic spreads between the quadratus lumborum and erector spinae muscles, potentially providing analgesia to the lower abdominal wall.^101^ A case series suggests that QIPB may provide effective postoperative analgesia following living-donor nephrectomy.^102^ Further prospective studies are required to establish its efficacy, safety, and performance relative to established techniques in renal transplantation (Table 3).

## Conclusion

Regional anaesthesia is increasingly used in liver and kidney transplantation to improve analgesia, reduce opioid requirements, and support recovery while maintaining hemodynamic stability. Neuraxial techniques may be feasible in selected patients, but are limited by coagulation disturbances and cardiovascular risk, particularly during liver transplantation. Ultrasound-guided fascial plane blocks represent a safer and anatomically suitable alternative in high-risk populations. Further prospective comparative studies are needed to define optimal strategies and guide their integration into standardized transplant care pathways.

## Figures and Tables

**Table 1. Comparison of Neuraxial and Fascial Plane Blocks in Transplantation:** 

**Technique**	**Pros**	**Cons/risks**	**Clinical considerations**
**Neuraxial** **techniques** **(epidural/spinal)**	Provides superior analgesia, reduces opioid consumption, and attenuates surgical stress. Facilitates early GI recovery and improves respiratory function.	Risk of epidural hematoma due to dynamic coagulation abnormalities. Potential for profound hypotension impacting graft perfusion.	Feasible in carefully selected patients, particularly living donors. Requires real-time coagulation monitoring (e.g., TEG/ROTEM).
**Fascial** **plane** **blocks** **(US-guided)**	Favorable safety profile in high-risk populations with altered hemostasis. Performed in superficial planes where bleeding is easily managed. Preserves hemodynamic stability.	Primarily targets somatic pain; visceral coverage varies by technique. Evidence level is still evolving for some novel blocks.	Ideal for ERAS protocols and patients with significant cardiovascular vulnerability.

GI, gastrointestinal; ERAS, enhanced recovery after surgery; TEG, thromboelastography; ROTEM, rotational thromboelastometry; US, ultrasound

**Table 2. Summary of Regional Anaesthesia Techniques in Liver Transplantation:** 

**Technique**	**Population**	**Clinical efficacy & reported benefits**	**Evidence level/studies**	**Complications**
**Epidural** **analgesia**	Donors & recipients	Superior pain control and improved pulmonary function compared to IV PCA.	Extensive retrospective series (e.g., 685 recipients).	Risk of hematoma in coagulopathy.
**ESPB**	Donors & recipients	Reduced opioid consumption and lower pain scores. Earlier return of bowel function in pediatric cases.	Randomized controlled trials and feasibility studies.	None reported in transplant patients to date.
**QLB**	Recipients	Shorter extubation times and reduced intraoperative blood transfusion requirements.	Randomized trials and single-center retrospective studies.	Theoretical risk of organ injury or motor block.
**TAPB**	Donors & recipients	Effective somatic analgesia and potential reduction in extubation times.	Meta-analysis of three studies; several case series.	None reported.
**M-TAPA**	Donors & recipients	Reduces opioid use and side effects; performed in supine position.	Prospective randomized controlled study (donors).	None reported.
**EOIB**	Donors & recipients	Comparable to subcostal TAPB for lateral incisions.	Small series and randomized trials.	Theoretical risk of pneumothorax.

IV PCA, intravenous patient-controlled analgesia; ESPB, erector spinae plane block; QLB, quadratus lumborum block, TAPB: transversus abdominis plane block, M-TAPA: modified thoracoabdominal nerve block through perichondrial approach, EOIB: external oblique intercostal plane block, US: ultrasound

**Table 3. Summary of Regional Anaesthesia Techniques in Kidney Transplantation:** 

**Technique**	**Population**	**Clinical efficacy & reported benefits**	**Evidence level/studies**	**Complications**
**TAPB** **(+rectus** **sheath)**	Recipients	Analgesia comparable to epidural with fewer side effects.	Randomized non-inferiority trials.	None reported.
**Anterior** **QLB**	Donors & recipients	Superior pain control and reduced opioid consumption compared to ESPB or systemic analgesia.	Randomized controlled trials.	None reported.
**ESPB**	Donors & recipients	Effective opioid-sparing effects with a favorable safety profile.	Case reports and comparative studies.	None reported.
**QIPB**	Donors	Novel technique for lower abdominal wall analgesia.	Emerging evidence (case reports and small series).	None reported.

TAPB, transversus abdominis plane block; QLB, quadratus lumborum block; ESPB, erector spinae plane block; QIPB, quadro-iliac plane block
